# Improved Survival and Quality of Life Through an Integrative, Multidisciplinary Oncological Approach: Pathophysiological Analysis of Four Clinical Cancer Cases and Review of the Literature

**DOI:** 10.3389/fphar.2022.867907

**Published:** 2022-06-16

**Authors:** M. Berretta, A. Morra, R. Taibi, F. Monari, N. Maurea, M. Ippolito, U. Tirelli, F. Fiorica, L. Montella, G. Facchini, V. Quagliariello, M. Montopoli

**Affiliations:** ^1^ Department of Clinical and Experimental Medicine, University of Messina, Messina, Italy; ^2^ Integrative Medicine Research Group, IMRG, Noceto, Italy; ^3^ IRCCS SDN, SYNLAB Napoli, Naples, Italy; ^4^ Gruppo Oncologico Ricercatori Italiani, GORI-Onlus, Pordenone, Italy; ^5^ Radiotherapy Unit, Policlinico Di Sant'Orsola, University of Bologna, Bologna, Italy; ^6^ Division of Cardiology, Istituto Nazionale Tumori, Naples, Italy; ^7^ Department of Advanced Technologies, Nuclear Medicine and PET, “Cannizzaro” Hospital, Catania, Italy; ^8^ Tirelli Medical Center, Pordenone, Italy; ^9^ Department of Radiation Oncology and Nuclear Medicine, Verona, Italy; ^10^ ASL NA2 NORD, Oncology Operative Unit, “Santa Maria Delle Grazie” Hospital, Naples, Italy; ^11^ Department of Pharmaceutical and Pharmacological Sciences, University of Padova, Padova, Italy

**Keywords:** malignant mesothelioma, cholangiocarcinoma, breast cancer, treatment, cancer, integrative medicine, personalized medicine, medicinal mushrooms

## Abstract

**Objectives:** According to the National Cancer Institute, the integrative medicine (IM) approach to medical care combines standard medicine with complementary and alternative medicine practices that have proved safe and effective.

**Methods:** We describe the clinical cases of four patients with malignant pleural mesothelioma (MPM), diffuse malignant peritoneal mesothelioma (DMPM), intrahepatic cholangiocarcinoma, and breast cancer (BC) who received supportive treatment (ST) according to an IM approach after the failure of standard cancer treatments or the appearance of serious adverse events caused by antiblastic chemotherapy. The critical role of complementary drugs in reducing the side effects of cancer treatments and normalizing the white cell count is especially apparent in the case of the patient with metastatic BC, who experienced prolonged neutropenia.

**Results:** The IM approach was well-tolerated and had no adverse side effects. It improved the quality of life (QoL) of all patients and in two cases extended overall survival.

**Conclusion:** The extended clinical and instrumental response to IM of the patients with malignant mesothelioma and the improved health-related QoL and good tolerance of the ST demonstrated in all cases support the value of this approach in patients whose cancer therapies have failed but who show a good performance status. Our data require confirmation in a well-designed prospective clinical trial.

## Introduction

Health-related quality of life (HR-QoL) is a critical outcome measure in cancer management. It is a dual notion since in terminally patients it involves control of disease-related symptoms, such as pain and cancer-related fatigue (CRF), and of nutritional intake, whereas in patients receiving active cancer treatment [e.g., antiblastic chemotherapy (AC), immunotherapy (IMT), and radiotherapy (RT)] it means control of treatment-related side effects. Satisfactory results have widely been reported (İnci and İnci, 2020) and are best achieved by multidisciplinary teams. In this scenario, integrative medicine (IM) is a new care opportunity (Halil Güneş et al., 2020; Khazaei et al., 2020; Olçar and Karadağ, 2020; İnci and İnci, 2020). According to the US National Cancer Institute (NCI), the IM approach combines standard medicine with complementary and alternative medicine (CAM) practices that have proved safe and effective (PDQ Integrative et al., 2002; Berretta et al., 2017; Berretta et al., 2020c; Berretta et al., 2020a). IM tries to stress patient preferences and to address their mental, physical, and spiritual health.

**FIGURE 1:**
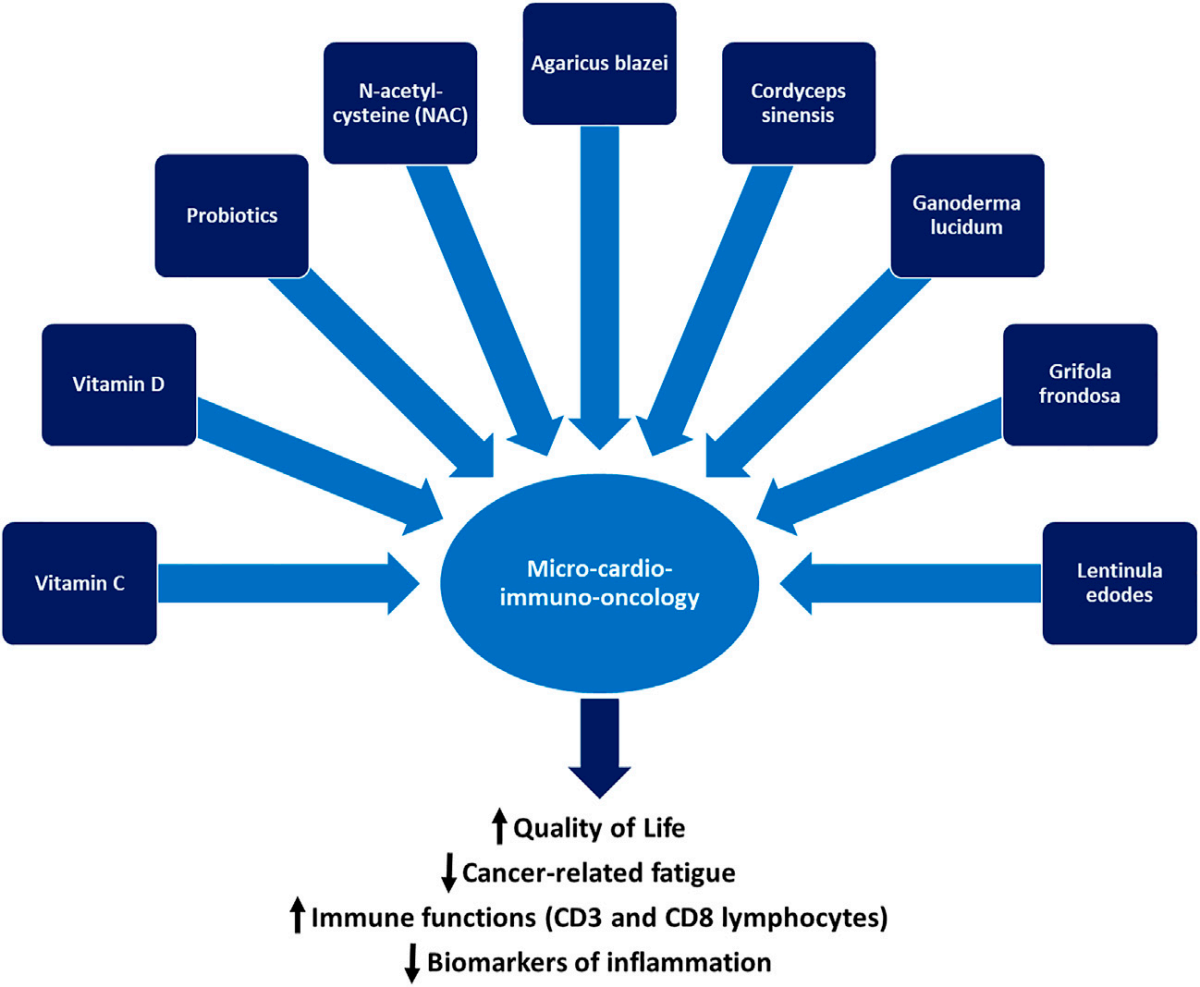
The micro-cardio-immuno-oncology axis and the mainstays of complementary and alternative medicine: vitamin C, vitamin D, probiotics, N-acetyl-cysteine, and selected medicinal mushrooms can improve the quality of life and immune function of patients with cancer and reduce cancer-related fatigue and the systemic concentration of pro-inflammatory biomarkers.

The biochemical basis for the clinical use of CAM in patients with cancer is its ability to improve the systemic biomarkers of inflammation and prognosis and to delay recurrence. Moreover, some CAM practices reduce the risk factors for cardiometabolic conditions such as diabetes, insulin resistance, metabolic syndrome, and visceral obesity, which involve a higher risk of cancer recurrence, heart failure, atherosclerosis, and overall mortality. Some of the anti-inflammatory effects of CAM are exerted through attenuation of the cytokine storm triggered by the NLRP3 and MYD88 pathways. As summarized in Figure 1, the “micro-cardio-immuno-oncology” axis, a deep network encompassing the microbiome, the cardiovascular and immune systems, and cancer pathways, is susceptible to several types of CAM, which has the ability to improve HR-QoL, CRF, immune function, and survival.

Crucially, in patients receiving active cancer treatment, HR-QoL preservation allows for completing AC, IMT, and RT cycles.

The four cases described below clearly demonstrate the safety and efficacy of an IM approach that was devised by a multidisciplinary team as an oncological approach and supportive treatment (ST) and shared with the patients.

Malignant mesothelioma (MM) is a rare, aggressive cancer arising from the mesothelium, the thin tissue that lines the lungs, chest wall, and abdomen. The major risk factors for MM are asbestos exposure and viral infection (e.g., Simian Virus 40, SV40). The most common histological subtypes are the epithelioid (60–80%), the sarcomatoid (20%), and the biphasic (Vita et al., 2021). The outcome of MM is closely cell type-dependent—for instance, epithelioid MM is less aggressive, even though long-term outcomes are still poor. The standard MM treatment is currently based on a multimodal approach, including induction AC (platin and pemetrexed), surgical resection, and sometimes RT, which is generally offered to patients with pleural MM, to young patients with a good Eastern Cooperative Oncology Group (ECOG) (Martin, 2021) performance status (PS), to patients with localized disease and to those with the epithelioid subtype (Vita et al., 2021). The clinical role of second-line AC for progressive or relapsed disease is still undefined, as no post-progression validated treatment has emerged. Recent advances in immunotherapy may provide a breakthrough in the future. In the meantime, poor clinical condition and PS and relapsed and resistant disease after treatment failure hamper therapeutic decision-making, since it is difficult to decide whether to offer second-line treatment or best supportive care (BSC).

iCCA is a rare, aggressive primary liver tumor characterized by a variety of clinical manifestations and by high incidence and mortality rates even after curative treatment with radical resection (Berretta et al., 2015; Xiaopei et al., 2019). Different types of treatments are available for these patients in relation to disease stage, PS, comorbidities, and age. Some are standard (surgery, AC, LRT, and RT), whereas others are being tested in clinical trials. The prognosis is usually poor.

Breast cancer (BC) is still the most commonly diagnosed cancer in women all over the world (Caputo et al., 2020). The majority of patients with metastatic BC experience recurrent metastatic disease after the primary treatment for earlier-stage BC, whereas only a small fraction present with *de novo* metastatic disease (Mallet et al., 2022). Metastatic BC is currently considered incurable. For this reason, the primary therapeutic goals in these patients are an extension of survival, maintenance of the HR-QoL, and palliation of symptoms.

Our four patients suffered from malignant pleural mesothelioma (MPM), diffuse malignant peritoneal mesothelioma (DMPM), intrahepatic cholangiocarcinoma (iCCA), and breast cancer (BC) which had been managed with first-line systemic AC (MPM, DMPM, and BC) or loco-regional treatment (LRT; iCCA). After treatment failure, they received a similar ST.

We report the results of ST administration to patients with limited chances of standard cancer treatment due to disease progression or the appearance of serious adverse events.

## Patients and Methods

The three patients received standard oncological treatment before beginning the ST, which had been devised by a multidisciplinary team according to international clinical guidelines. The SP consisted mainly of vitamins C (Berretta et al., 2020b) and D, probiotics, and a blend of medicinal mushrooms (Micotherapy U-care, ADV Reform srl, Noceto, Italy) (Roda et al., 2020). Medicinal mushroom extract mixture micotherapy has been registered by the Italian Ministry of Health as a dietary supplement (registration number 627 I.5.i.h.2/2020/627). It is produced from a hot water extract which is precipitated with ethanol and subsequently freeze-dried. This tablet preparation contains a mixture of *Ganoderma lucidum, Grifola frondosa, Agaricus blazei, Cordyceps sinensis*, and *Lentinula edodes*, in equal amounts i.e. 300 mg each per tablet. One tablet, therefore, contains total polysaccharides >30% and 1.3–1.6 beta-glucans > 15%.

Probiotics (Acticolon) are a freeze-dried probiotic preparation containing 2 × 10^9^ cfu microorganisms/day strains of lactic acid bacteria (*Lactobacillus rhamnosus* LRH11, *Lactobacillus acidophilus* LA5, and *Bifidobacterium bifidum* BB12), microcrystalline cellulose, stearic acid, magnesium stearate, and vegetable capsule (hydroxypropyl methylcellulose), silicon dioxide (registration number 3587 I.5. i.h.2/2018/3587). Patients received ample information about the proposed ST and signed the informed consent. Data collection and analysis of these cases were in line with the principles of the Declaration of Helsinki. Patients’ clinical characteristics are reported in Table 1.

**TABLE 1:** Patients’ clinical characteristics.

Patients characteristics	Case 1	Case 2	Case 3	Case 4
Age at diagnosis	64	55	76	38
Gender	Male	Male	Female	Female
PS (ECOG)	0	0	1	0
Histology	MPM	DMPM	CCA	BC
TNM stage at diagnosis	IV	IIIB	III-B	IA
First-line treatment	AC (DDP/pemetrexed)	AC (JM8/pemetrexed)	TARE (Y90)	PTP**
Response to treatment	PR + CR	CR	PR	PR
Second-line/maintenance treatment	Pemetrexed	NA	TACE (ADM)	TDM-1
Response to treatment	PR + CR	NA	PR	SD
Third or more line treatment	NA	NA	TACE (ADM + DDP)	-
Response to treatment	NA	NA	SD	-
Toxicities	Cutaneous infections fatigue	Acute renal failure fatigue	Vomiting fatigue	-
Total treatment cycles	11	6	4	45
Total months from diagnosis/metastatic disease	29	24	54	16 years
Total months from ST	17	12	5	1
Response to ST	PR + improved QoL	SD + improved QoL	Improved QoL	NA

Legend: PS: performance status; MPM: malignant pleural mesothelioma; DMPM: diffuse malignant peritoneal mesothelioma; CCA: cholangiocarcinoma; BC: breast cancer; DDP: cisplatin; JM8: carboplatin; PR: partial remission; CR., complete remission; ADM: adriamycin; TARE: Trans-arterial radioembolization yttrium-90 (90Y); TACE: trans-arterial chemoembolization; TDM-1: trastuzumab emtansine; PTP: pertuzumab, trastuzumab, paclitaxel; ** first metastatic line; NA: not applicable; ST: support treatment; QoL: quality of life.

CASE 1 Malignant pleural mesothelioma

This 64-year-old man suffered from metastatic MPM. He was admitted to the emergency room in May 2019 due to dyspnea and pain in the right chest wall. The chest x-rays and CT scan disclosed right pleural effusion and a suspicious osteolytic lesion on the right 9^th^ rib. The patient underwent partial right parietal pleurectomy and pleurodesis with medical graded talc using a video-assisted thoracoscopy surgery approach. There were no postoperative complications. The histological examination revealed a pleural epithelial mesothelioma, whereas immunohistochemistry (IHC) showed that the tissue was positive for calretinin, CK5/6, and D2-40 and negative for EpCAM and TTF1. Perioperative staging with total body CT and CT/PET fdg scans demonstrated mediastinal lymph node and left adrenal gland metastases. From August to December 2019, the patient received six cycles of pemetrexed/cisplatin AC as a first-line treatment and achieved partial (PR) and complete remission (CR) respectively of his lymph node and adrenal gland metastases. The AC was well-tolerated and there were no serious adverse events. From January to August 2020, the patient was scheduled to receive six cycles of pemetrexed single-agent AC (q28) as the maintenance treatment (Bearz et al., 2008). Eventually, he developed marked CRF, (Giacalone et al., 2013; Giacalone et al., 2012) lack of appetite, and recurrent erysipelas infection of the lower limbs. A new CT/PET fdg scan showed a stable disease (SD). Given the total number of AC cycles already administered, his objective response, the adverse events, and the HR-QoL, we decided together with the patient to suspend the AC and begin follow-up and ST, whose primary goals were to manage the erysipelas infection and to improve HR-QoL. The ST consisted of antibiotics and oral vitamin C, vitamin D (in relation to its serum level), probiotics, and a blend of medicinal mushrooms (Micotherapy U-care care) (see Table 2 for dosage). The use of the mushroom blend was supported by clinical evidence obtained from cancer patients (Roda et al., 2020; Del Buono et al., 2016; Barbieri et al., 2017; Rossi et al., 2018). The ST was well tolerated, there were no new infections and the HR-QoL improved quickly. The patient returned to work. In February 2021, a new CT/PET fdg scan, performed 6 months from AC withdrawal, documented a PR of the disease. The ST was continued until May 2021, when a new CT/PET fdg scan showed a minimal progression of disease (PD) with the involvement of a lumbar aortic lymph node. Considering the single site, in June 2021 the patient underwent RT (7Gy in five fractions). The most recent CT/PET fdg scan shows CR. Notably, the time to progression (TTP) was 22 months, 13 months while receiving AC, and 9 months while receiving ST. Thirty-two months from the diagnosis, the patient is alive. The ST is well-tolerated and has induced no serious adverse events. The patient’s HR-QoL is excellent and his PS (0) is good.

**TABLE 2:** Support treatment administered as an IM approach.

Natural compound	Total concentration	Dosage	Duration
Vitamin C	1,000 mg	1 tab/day, 21 days/month	17 months
Vitamin D	50,000 IU	1 oral vial/month	17 months
Lactobacillus rhamnosus LRH11		2 tabs/day 21, days/month	17 months
Lactobacillus acidophilus LA5			
Bifidobacterium bifidum BB12			
Agaricus blazei	300 mg	2 tabs/day	17 months
Cordyceps sinensis	300 mg		
Ganoderma lucidum	300 mg		
Grifola frondosa	300 mg		
Lentinula edodes	300 mg

CASE 2 Diffuse malignant peritoneal mesothelioma

The second case involved a 56-year-old man with DMPM. In July 2019, he underwent exploratory laparotomy due to ascites and peritoneal nodules. The histological examination identified peritoneal epithelial mesothelioma that was positive for CK5/7 and calretinin and negative for CK20 and CDX2. There were no postoperative complications. Postoperative staging, documented by total body CT and CT/PET fdg scans, demonstrated a diffuse peritoneal involvement. From August 2019 to January 2020, the patient received first-line pemetrexed/carboplatin AC (6 cycles) and achieved CR, as documented by a CT/PET fdg scan. The AC was well tolerated without serious adverse events and the patient began the follow-up. In April 2020, he experienced PD complicated by acute renal failure (ARF) with an estimated glomerular filtration rate (eGFR) of 13 ml/min, diffused abdominal pain due to frozen pelvis, and loss of appetite and weight. Given his clinical condition and the fact that he could receive no further AC cycles, we decided, together with the patient to begin ST. This consisted of hydration therapy, oral N-acetyl-cysteine (NAC), vitamin D, intravenous (iv) vitamin C, probiotics, and micotherapy U-care (see Table 3 for dosage). The primary treatment goals were to address the ARF and improve symptoms and HR-QoL. The ST was well-tolerated. The HR-QoL improved quickly as did renal function (eGFR, from 13 to 23 ml/min), the abdominal pain disappeared and the patient began eating again. In August 2020, a new CT scan showed SD, which remained unchanged until June 2021, when PD was documented. The patient died in July 2021 due to PD. The most notable outcomes of this case with aggressive cancer are an OS of 24 months and a TTP of 14 months from ST inception.

**TABLE 3:** Support treatment administered as an IM approach.

Natural compound	Concentration	Dosage	Duration
Vitamin C	1,000 mg	3 vials/day	14 months
Vitamin D	50,000 IU	1 oral vial/month	14 months
N-acetyl-cysteine	600 mg	1 tab/day	10 months
Lactobacillus rhamnosus LRH11		2 tabs/day, 21 days/month	10 months
Lactobacillus acidophilus LA5			
Bifidobacterium bifidum BB12			
Agaricus blazei	300 mg	2 tabs/day	14 months
Cordyceps sinensis	300 mg		
Ganoderma lucidum	300 mg		
Grifola frondosa	300 mg		
Lentinula edodes	300 mg

CASE 3 Intrahepatic cholangiocarcinoma

This patient was a 76-year-old woman with iCCA. Her family history included the death of three brothers from bile duct cancer. In June 2017, she underwent a liver biopsy due to right abdominal pain, weight loss, dyspeptic symptoms, and CT evidence of liver lesions. Histopathological examination demonstrated a poorly differentiated carcinoma that was positive for pancreatin and CK7 and negative for TTF1, hepatocyte, and CK20 and was consistent with upper gastrointestinal tract biliary-pancreatic origin and iCCA. The blood examination evidenced an alpha-fetoprotein (αFP) value of 450 IU/ml (range, 0–15) and a lactate dehydrogenase (LDH) value of 512 (range, 230–460), which suggested a clinical diagnosis of iCCA. In consideration of her age, comorbidities (hypertension), disease stage (IIIB TNM AJCC), and the poor sensitivity of iCCA to AC, in November 2017 the patient received trans-arterial radioembolization (TARE) with yttrium-90 (Y90) resin microspheres, an emerging local treatment option for iCCA (Paprottka et al., 2021). There were no serious adverse events. The patient achieved PR, as demonstrated by a CT scan performed at 3 months. Her αFP value fell significantly (from 450 to 27). The patient began a quarterly follow-up. In August 2019, after 21 months of first-line treatment, a new CT scan showed local PD. After multidisciplinary consultation, we proposed two cycles of trans-arterial chemoembolization (TACE) with adriamycin (ADM), which were administered between August and September. The treatment was well-tolerated, the ECOG PS was 0 and in December 2019 the CT scan showed a PR. PD was documented in June 2020, 6 months into the second-line treatment. A month later the patient received the third cycle of TACE with ADM and cisplatin, which induced moderate and persistent adverse events including nausea, vomiting, weight loss, and CRF. In January 2021, 5 months from the third cycle of TACE, a new CT scan showed local PD. The patient refused palliative single-agent chemotherapy, due to the previous adverse events. We, therefore, decided to wait and see and began follow-up, based on her stable clinical condition and her PS of 1. In August 2021, a further local PD was documented by CT and abdominal ultrasound scans. Her αFP rose to 192 IU/ml; the abdominal pain and loss of appetite and weight returned and CRF was the most evident symptom; the ECOG PS was 2. Considering the relevant factors (PD, clinical condition, previous treatments, refusal to undergo a new TACE, and age), we proposed to the patient to begin ST, to reduce her CRF and improve her HR-QoL. The ST consisted of oral administration of NAC, vitamins C and D, probiotics, and micotherapy U-care (see Table 4 for dosage) and was well-tolerated. There were no serious adverse events and the QoL improved quickly; the CRF decreased significantly and a moderate weight gain was observed. In January 2022, an abdominal ultrasound scan showed a liver PD at S_8_ (52 vs. 70 mm), an SD at S_4_ (63 vs. 65 mm), and three new liver lesions <1 cm. The αFP value rose to 200 IU/ml with normal serum bilirubin and AST/ALT. The patient’s PS was 1. Her clinical condition deteriorated and she was admitted to the hospital, where she died in January 2022 due to the rapid decline of her clinical conditions.

**TABLE 4:** Support treatment administered as IM approach.

Natural compound	Concentration	Dosage	Duration
Vitamin C	1,000 mg	1 tab/day 21, days/month	12 months
Vitamin D	25.000 IU	2 times monthly	14 months
N-acetyl-cysteine	600 mg	1 tab/day	4 months
Lactobacillus rhamnosus LRH11		2 tabs/day, 21 days/month	4 months
Lactobacillus acidophilus LA5			
Bifidobacterium bifidum BB12			
Agaricus blazei	300 mg	2 tabs/day	4 months
Cordyceps sinensis	300 mg		
Ganoderma lucidum	300 mg		
Grifola frondosa	300 mg		
Lentinula edodes	300 mg

Case 4 Metastatic breast cancer with prolonged neutropenia after Comirnaty vaccine administration

**FIGURE 2:**
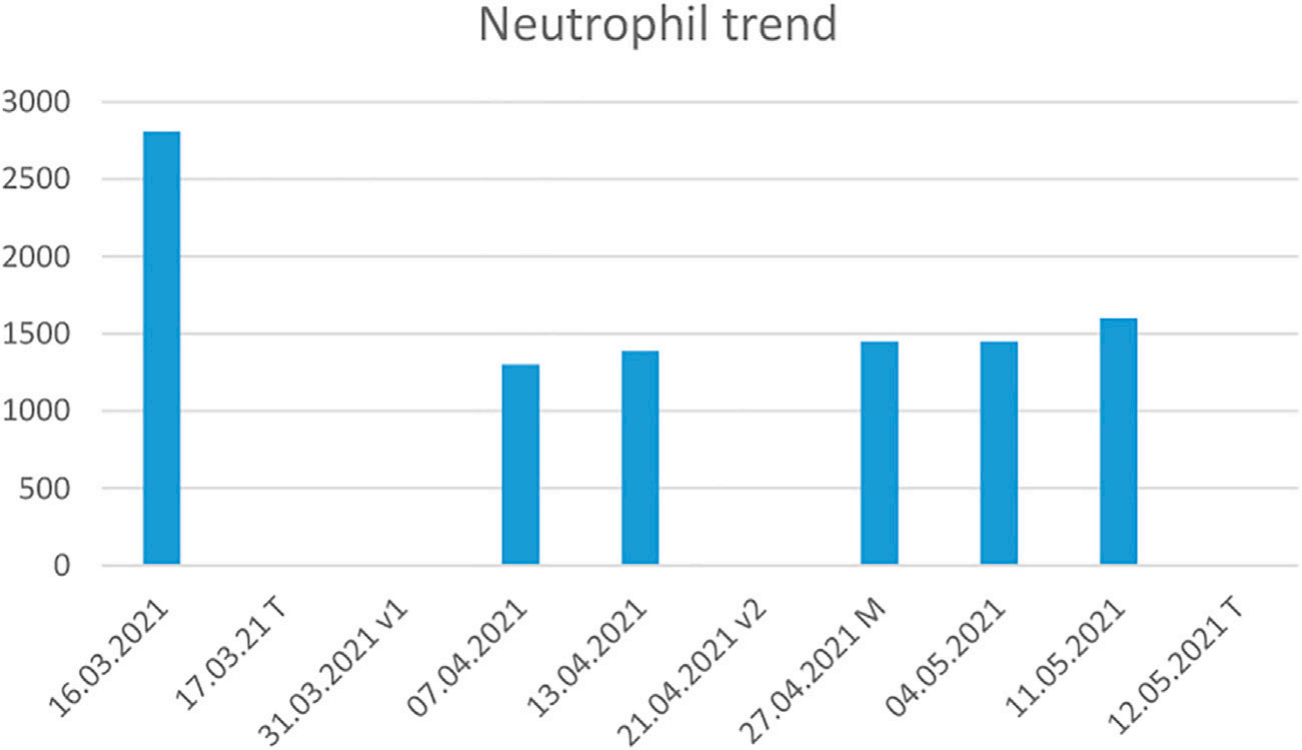
Graphical representation of the neutrophil trend, which declined after the first vaccine dose and whose persistently a low level required extending the interval between antiblastic treatments to nearly 2 months. T: treatment administration, v1: first vaccine dose, v2: second vaccine dose, M: mushrooms integration.

The fourth patient was a 62-year-old woman with lymph node and lung metastatic disease from a primary hormone-receptor and HER2 positive BC that had been diagnosed and surgically removed in 1997. Eight years later (May 2005), lung metastases were discovered by CT scan and primarily treated with radical stereotactic radiotherapy. At further PD, in January 2017, she was treated with the standard first-line AC treatment for metastatic disease (pertuzumab and trastuzumab) for about 2 years. In February 2018, the patient began the second-line treatment with trastuzumab emtansine, which is ongoing and still ensures disease control. In January 2021, the worldwide Covid-19 pandemic involved the launch of a massive vaccination campaign to protect the population, especially fragile and oncological patients, from the adverse outcomes of Covid-19 virus infection. The most widely used vaccines were based on messenger ribonucleic acid (mRNA) molecules, which stimulate host cells to synthesize the Spike surface protein of Coronavirus. A large body of data has demonstrated the safety of such vaccines (Faermann et al., 2021; Seban et al., 2021). Our patient received the two doses of Pfizer-BioNTech (COMIRNATY) BNT162b2 mRNA between March and April 2021, at the correct 3-weeks interval. Following the second dose, the patient—who had never needed treatment postponement or dose reduction—experienced prolonged grade-2 neutropenia (see Figure 2) from which she recovered only in May 2021 after administration of micotherapy U-care for 15 days (see Table 5). The ST was administered for a month. From then on she has tolerated AC without further episodes of neutropenia.

This case is not comparable to those of the first three patients, either in terms of prognosis or of available therapeutic options, which in this patient are very limited in number and effectiveness. BC is largely a curable tumor that can be managed with a powerful armamentarium of several and ever-increasing lines of treatment even in patients with advanced/metastatic disease. However, other issues can be found in the management of these prolonged histories of succeeding therapies. In particular, clinicians may deal with phases of psychological refusal and adverse events related to psycho-organic factors like asthenia that are not easily relieved by available supportive care. The control of adverse events is essential to achieve and maintain a good HR-QoL and optimize patient compliance. In this case, AC combined with the mRNA Covid-19 vaccine induced a prolonged phase of neutropenia that was probably due to the immune reaction to lymphocyte populations concentrating at peripheral drainage sites. This phenomenon may reflect the relative reduction in peripheral blood and the atypical figures found especially in BC patients following Covid-19 vaccination (Faermann et al., 2021; Seban et al., 2021).

Micotherapy has recognized properties in attenuating the side effects of anticancer treatments, specifically AC-induced neutropenia (Liu et al., 2008; Ito et al., 2009; Jiang et al., 2017; Lei et al., 2020). The mushroom blend is rich in polysaccharides and B-glucans, which have well-documented immunomodulatory properties. Although their heterogeneous composition and the lack of funds hamper basic as well as clinical research on these types of ST, work in this area would provide valuable insight into the role and properties of micotherapy and help its wider use as an ST.

**TABLE 5:** Support treatment administered as IM approach.

Natural compound	Concentration (mg)	Dosage	Duration
Agaricus blazei	300	2 tabs/day	4 months
Cordyceps sinensis	300		
Ganoderma lucidum	300		
Grifola frondosa	300		
Lentinula edodes	300		

## Discussion

Integrative medicine is a novel approach to medical care that combines standard medicine with CAM practices that have been shown to be safe and effective.

The four clinical cases described herein provide typical examples of an IM approach that was devised by a multidisciplinary team and shared with the patient. All four patients, who suffered from metastatic MPM and DMPM, locally advanced iCCA, and metastatic BC, had a poor prognosis and their median OS was, respectively, 15.3 months (Bearz et al., 2008), 11.5 months (Serio et al., 2017), 12.5 months (Mavros et al., 2014), and 55 months (Mallet et al., 2022). After the failure of oncological treatment, these patients experienced a progressive deterioration of the clinical condition and PS, and the only available therapy is often palliative treatment. The rationale for administering an ST consisting of vitamins C and D, probiotics, and medicinal mushrooms was based on clinical evidence reported in the literature and on the guidelines of the NCI and the National Center for Complementary and Integrative Health (NCCIH) (PDQ Integrative et al., 2002), which consider IM as a valid ST strategy after the failure of standard cancer treatments and/or the occurrence of serious adverse events. IM-based ST is closely patient-tailored since it depends on a variety of parameters such as age, PS, comorbidities, cancer-related symptoms, treatment failure, AC cycles received, adverse events, and terminal illness. The ST drugs/supplements proposed to our patients were based on their clinical features and disease characteristics. Vitamin C improves HR-QoL and attenuates cancer-related side effects (Berretta et al., 2020b). The most important clinical benefits are provided by iv administration but in the patients with MPM and iCCA oral vitamin C did enhance HR-QoL (Berretta et al., 2020b). Our STs were safe and effective, they did not induce adverse events and significantly improved HR-QoL; moreover, they extended OS in the two patients with MM.

The use of vitamin D is supported by observational studies which have demonstrated that it is associated with longer survival in cancer patients (Imran Ali Shah, 2020; İnci et al., 2020; Gnagnarella et al., 2021). Moreover, there are reasonable mechanisms for its effect in reducing tumor invasiveness and propensity to metastasize and in influencing immunomodulatory properties (Jiang et al., 2017; Imran Ali Shah, 2020; İnci et al., 2020; Gnagnarella et al., 2021) that may contribute to reducing metastatic disease and fatal cancer.

Probiotics are live microorganisms that are known to attenuate the side effects of AC and in general to restore intestinal eubiosis and nutrient absorption, which are the key mechanisms to improve outcomes and HR-QoL. Administered in adequate doses, they confer benefits such as improved immune function and support for the competitive exclusion of pathogens (Deleemans et al., 2021). In oncological settings, they may help to maintain the balance of the intestinal microbiota, to reduce potential pathogenic bacterial infection, improve bowel regularity, and restore homeostasis to the intestinal microbiota after AC (Barbieri et al., 2017; Rossi et al., 2018; Garau et al., 2021).

The role of medicinal mushroom blends is more intriguing. According to the NCI and the NCCIH, they can improve HR-QoL and immunomodulation. Moreover, they have been approved as adjuncts to standard cancer treatment in Japan and China more than 30 years ago and have an extensive history of safe clinical use alone or combined with RT or AC (Roda et al., 2020; Barbieri et al., 2017; Nowakowski et al., 2021). The different blends produce numerous bioactive compounds that influence several cancer-related pathways, often synergistically, also by modulating the cellular targets involved in cell proliferation, survival, and angiogenesis (Barbieri et al., 2017; Rossi et al., 2018; Garau et al., 2021; Nowakowski et al., 2021).

Medicinal mushrooms exert immune enhancing activities through the stimulation of the growth and differentiation of lymphocytes (Moradali et al., 2007; Vanneman and Dranoff, 2012). For example, Turkey tail extract is able to increase CD8^+^ T cells and CD19 ^+^ B cells in breast cancer patients (Martínez-Montemayor et al., 2011). Reishi increases significantly IFN-γ, IL-2, IL-6, and NK cells (CD56 ^+^ cells) levels in lung cancer patients thereby increasing potentially immune therapies (Gao et al., 2003). Other studies indicate that the mushrooms reishi, cordyceps, agaricus, and maitake reduce TH-2 cytokines with anti-inflammatory and immune-enhancing functions (Kar Mahapatra et al., 2011). The clinical outcomes of medicinal mushrooms still to be studied in a deeper manner; however, it has been clarified that protein complexes, polysaccharides, and β-glucans of medicinal mushrooms reduce NF-kB expression in APC cells, increase the synthesis and release of INF-γ from NK cells, and TH-1 pathways which lead to the activation of macrophages and cytotoxic lymphocytes with antitumor properties (Borchers et al., 2004; Guggenheim et al., 2014; Zhao et al., 2020).

Here, every supplement/drug included in IM approaches has a different beneficial role and action that often complements those of the other treatment constituents. We believed that these supplements exert a synergistic activity, for instance on the gut microbiota. There is evidence that gut microbiota dysbiosis can trigger inflammatory signaling pathways that affect the intestinal and extra-intestinal immune function and contribute to carcinogenesis and cancer progression (Toor et al., 2019; Rizzetto et al., 2018). Carcinogenesis is an inflammatory process where the microbiota appears to be involved both directly and through indirect mechanisms, via the immune pathways. The primary goals of the ST administered to our patients were to improve HR-QoL and help the immune system contrast disease progression. The good TTP and OS outcomes of the patients with MPM and DMPM were frankly unexpected.

According to the English literature, about half of cancer patients already combine CAM remedies with oncological treatments without informing their physician (Berretta et al., 2020c). We feel that this decision should be made by the medical staff. Nutraceuticals fully fall into the category of CAM with interesting implications in oncology and cardioncology (Quagliariello et al., 2018). Nutraceuticals are extracts of medicinal plants or complexes of natural bioactive able to reduce the metabolism of cancer cells by inhibiting the synthesis and release of cytokines, chemokines, and growth factors associated with apoptosis escape and cancer cell survival/angiogenesis (de Mejia and Dia, 2010). Nutraceuticals include curcuma longa, artichoke, milk thistle, rhodiola rosea, berberis, boswellia, and others. Several clinical studies have shown that nutraceuticals are able to reduce cancer and cardiovascular risk factors through anti-inflammatory activities (Barbarisi et al., 2019; Balakrishna and Kumar, 2015). However, their use must always be encouraged with the supervision of clinicians, avoiding self-administration, for possible drug-plant interactions that can reduce the anticancer efficacy and cancer outcomes (Yeung et al., 2018).

In conclusion, the extended clinical and instrumental response documented in the two patients with MM and the improved HR-QoL and good ST tolerance observed in all cases support the value of administering IM to patients whose oncological therapies have failed but who have a good PS.

Sometimes the patients themselves answer the question, choosing an alternative, and/or complementary treatment to obtain better results also in terms of HR-QoL and we firmly believing that this assessment is up to the healthcare staff.

CAM, adopted as an ST by a multidisciplinary team, is a major care opportunity for patients with cancer and for frail (elderly, HIV-positive) individuals (Berretta et al., 2010; Di Benedetto et al., 2011; Zanet et al., 2011). Clearly, our data need to be confirmed in a well-designed prospective clinical trial.

## Data Availability

The raw data supporting the conclusion of this article will be made available by the authors, without undue reservation.
